# Barriers to treatment optimization and achievement of patients’ goals: perspectives from people living with rheumatoid arthritis enrolled in the ArthritisPower registry

**DOI:** 10.1186/s13075-019-2076-7

**Published:** 2020-01-07

**Authors:** Kelly Gavigan, W. Benjamin Nowell, Mylene S. Serna, Jeffrey L. Stark, Mohamed Yassine, Jeffrey R. Curtis

**Affiliations:** 1 Global Healthy Living Foundation, Upper Nyack, NY USA; 2 UCB Pharma, Smyrna, GA USA; 3 University of Alabama at Birmingham, Birmingham, AL USA

**Keywords:** Rheumatoid arthritis, Treatment goals, Real-world data, RAPID3, Patient-reported outcomes

## Abstract

**Background:**

Few studies have investigated patients’ own treatment goals in rheumatoid arthritis (RA). The objective of this real-world, cross-sectional study of US patients with RA was to identify factors that patients believed influenced their physician’s treatment decisions. Secondary objectives included reasons patients tolerated sub-optimal disease control and their perceived barriers to treatment optimization.

**Methods:**

Eligible participants were enrolled in the ArthritisPower registry, ≥ 19 years, had physician-diagnosed RA, unchanged treatment within 3 months of baseline, prior/current disease-modifying antirheumatic drug treatment (DMARDs), and computer/smartphone access. In December 2017, participants completed Patient-Reported Outcomes Measurement Information System-Computerized Adaptive Tests (PROMIS-CAT) for pain interference, fatigue, sleep disturbance, and physical function. Routine Assessment of Patient Index Data 3 (RAPID3) provided disease activity scores (0–30). Participants completed an online survey on barriers to treatment optimization, including self-perception of disease compared to RAPID3/PROMIS scores.

**Results:**

A total of 249 participants met inclusion criteria and completed the survey. Mean age (SD) was 52 (11) years, and the majority were female (92%) with high RAPID3 disease activity (175/249 [70%]; median score 18). The main reason participants did not change treatment was their physician’s recommendation (66%; *n* = 32). Of participants with high RAPID3 disease activity, 66 (38%) were offered a treatment change; 19 (29%) of whom declined the change. Most participants who intensified treatment did so because their symptoms had remained severe or worsened (51%; *n* = 65); only 16 (25%) participants intensified because they had not reached a specified treatment goal. Among participants who self-reported their disease activity as “none/low” or “medium” (*n* = 202; 81% of cohort), most still had RAPID3 high disease activity (137/202 [68%]; score > 12). Most PROMIS scores showed moderate agreement with participants’ self-assessment of health status, in contrast to RAPID3 (weighted kappa: 0.05 [95% CI − 0.01, 0.11]).

**Conclusions:**

Most participants trusted their rheumatologist’s treatment decisions and prioritized their physician’s treatment goals over their own. Patients should be encouraged to share their treatment goals/expectations with their rheumatologist, in line with the treat-to-target approach. RAPID3 may be inappropriate for setting patient-centric treatment goals given the poor agreement with self-reported disease activity; most PROMIS scores showed better alignment with patients’ own assessments.

## Background

Over the last decade, treatment for rheumatoid arthritis (RA) has improved dramatically, in part due to the treat-to-target (T2T) approach recommended by the American College of Rheumatology [1, 2]. Rheumatologists are encouraged to involve their patients in setting treatment targets (e.g., remission, or at least low disease activity [LDA]) as part of the T2T approach. Validated composite measures of disease activity (e.g., Clinical Disease Activity Index [CDAI], Disease Activity Score [DAS], 28-joint Disease Activity Score [DAS28], or Simplified Disease Activity Index [SDAI]) should be used every 1–3 months and therapy adapted, if necessary, to achieve the agreed target. After achieving low disease activity or remission, disease activity should be assessed every 6 months to confirm whether achievement of the treatment target was sustained and whether further therapy adjustments are required [1, 2]. This approach has been shown to improve short- and long-term clinical and radiographic outcomes in RA [3, 4]. However, adherence to T2T guidelines in the U.S.A. is poor [5, 6]. Rheumatologists have identified irreversible joint damage and patient-driven undertreatment as the main barriers to optimal treatment in previous studies [1]. However, there are limited research studies that attempt to understand treatment goals from the patients’ perspective, including barriers to meeting treatment targets and factors that lead patients to tolerate sub-optimal disease control. Patient reluctance to adjust their treatment has been reported as a barrier to meeting T2T in the U.S.A. Corrona and TRACTION behavioral intervention trials (52% of patients and 37% of visits, respectively) [5, 7]. The patients’ perspective has become an increasingly important outcome assessment in RA [8]. Emphasis is placed on understanding patients’ self-perception of disease improvement and disease-related limitations [8].

The objective of this study was to identify the factors that people with RA believed had influenced their physician’s treatment decisions. The secondary objectives were to identify reasons why people with RA tolerated sub-optimal control of their disease (failure to achieve the recommended RA treatment goals of at least LDA), and their perceived barriers to treatment optimization. Participants were asked questions about their treatment goals, and any barriers that they believed prevented treatment intensification (e.g., starting or switching a biologic), particularly those that are potentially modifiable.

## Methods

### Study design and participants

We conducted an observational, cross-sectional study of participants enrolled in the ArthritisPower registry, a patient-powered research network for patient-centered outcomes research, comprised of adult individuals with arthritis or other rheumatologic conditions. ArthritisPower is a partnership of the CreakyJoints arthritis patient community, researchers at the University of Alabama at Birmingham, and the Global Healthy Living Foundation, a patient advocacy organization [9].

ArthritisPower members provided electronic consent to participate in the registry and were asked to opt-in if they wished to participate in the survey. All survey participants identified themselves as patients with RA. An email invitation with a unique link was sent to potentially eligible ArthritisPower members (December 2017) to ensure that members could only take the survey once and to track survey completion. Participants with incomplete surveys were sent a follow-up email. Eligible participants were ≥ 19 years of age, resided in the U.S.A., had physician-diagnosed RA, had not changed their RA treatment within 3 months prior to taking the survey, were currently receiving disease-modifying antirheumatic drug (DMARD) treatment or had previously taken DMARDs, and had access to a computer or smartphone.

### Survey content

Eligible participants were asked to rate their level of agreement with statements about their attitudes and knowledge of RA treatments. Participants completed four patient-reported outcome (PRO) measures included in the Patient-Reported Outcomes Measurement Information System – Computerized Adaptive Tests (PROMIS-CATs): Pain Interference, Fatigue, Sleep Disturbance, and Physical Function [10]. Participants also completed the Routine Assessment of Patient Index Data 3 (RAPID3; 0–30 scale) questionnaire as a patient-reported measure of disease activity [11]. Participants were then asked to complete a custom online survey concerning barriers to treatment optimization, which was developed collaboratively by the researchers and patient advocacy partners on the study team (Additional file 1: Table S1). Intensification of treatment was defined as an increased dose or frequency of administration of current medication, or the addition of, or switch to, a new medication. As part of this survey, participants were also asked to describe their overall RA disease activity in the 7 days prior to completing the survey as “none/low,” “medium,” or “high.”

### Statistical analysis

Participants were classified into three groups based on whether they were offered a treatment change by their treating rheumatologist at their most recent office visit, and whether, or not, that change was accepted by the participant, to create three mutually exclusive categories: change not offered, change offered and accepted, and change offered and rejected. Participants were also stratified by disease activity, assessed using the traditional cut-off points for RAPID3 (high > 12, moderate > 6 to ≤12, low > 3 to ≤ 6, and remission 0 to ≤ 3) [11].

A subgroup analysis considered participants with high disease activity (RAPID3 score > 12) at one or more additional time points in the 12 months prior to baseline and were therefore considered to have persistent high disease activity. Prior disease activity was determined by previous RAPID3 assessments collected with the ArthritisPower app between December 2016 and December 2017; assessments taken specifically for the survey were excluded. If multiple assessments were conducted during that time, the average of the scores was used.

Weighted kappa statistics with 95% confidence intervals (CI) were performed to determine if participants’ self-description of disease activity (“none/low,” “medium,” “high”) was correlated with their RAPID3 category (remission/low, moderate, or high disease activity). Weighted kappa statistics were also performed to determine whether participants’ self-description of pain interference, physical function, fatigue, and sleep disturbance (“none/low,” “medium,” “high”) were correlated with the corresponding PROMIS measurement (low, medium, high) [12]. Chi square tests and analysis of variance (ANOVA) tests were used to summarize categorical and continuous variables, respectively. All *p* values are nominal and should be interpreted in an exploratory manner. All data analyses were conducted using SPSS Version 25 (Armonk, NY, USA) and SAS Version 7.1 (SAS Institute Inc., Cary, NC, USA).

## Results

### Participant disposition and baseline characteristics

A total of 5541 patients were enrolled in the ArthritisPower RA registry, as of December 15, 2017 (Additional file 1: Table S2). Of whom 3191 patients were sent the survey via email between December 08, 2017–December 21, 2017. A total of 1006 patients opened the email, 433 patients clicked the link and 303 patients responded. Of the 303 respondents, 249 participants met the inclusion criteria and completed the survey. Baseline characteristics were similar between participants in high disease activity (*n* = 175, 70%; median RAPID3 score [IQR]: 18 [15–20]) and those not in high disease activity (Table 1) (moderate/low/near remission: *n* = 74, 30%). Almost all (89%) participants were currently receiving treatment with biologic and/or non-biologic DMARDs; the remainder had received DMARD treatment prior to baseline (all eligible participants had been treated with DMARDs at some point since RA diagnosis). PROMIS measurements of pain interference, fatigue, and sleep disturbance were higher in participants in high disease activity, and lower for physical function, when compared to participants not in high disease activity (Table 1).

**Table 1:** Baseline participants’ demographics by disease activity stratification, as measured by RAPID3 (*N* = 249)

Mean (SD) unless otherwise specified	All survey participants (*N* = 249)	High disease activity (*n* = 175)	Moderate/low disease activity or near REM (*n* = 74)	*p* value^a^
Age, years	51.7 (11.0)	50.9 (10.6)	53.7 (11.5)	0.06
Females, *n* (%)	229 (92.0)	163 (93.1)	66 (89.2)	0.29
Ethnicity, white, *n* (%)	225 (90.4)	154 (88.0)	71 (96.0)	0.05
Time since diagnosis, years (SD)	11.0 (9.5)	10.8 (9.3)	11.5 (10.1)	0.62
Some college education or above, *n* (%)	215 (86.4)	150 (85.7)	65 (87.8)	0.66
Full-time employment, *n* (%)	83 (33.3)	58 (33.1)	25 (33.8)	0.92
Private insurance, *n* (%)	154 (61.9)	112 (64.0)	42 (56.8)	0.28
Current RA therapy, *n* (%)				
Non-biologic DMARDs only	72 (28.9)	54 (30.9)	18 (24.3)	0.30
Biologic DMARDs	150 (60.2)	106 (60.6)	44 (59.5)	0.87
Steroid/NSAID/other/no treatment^b^	27 (10.8)	15 (8.6)	12 (16.2)	0.08
Patient-reported outcomes, median (IQR)				
RAPID3 (0–30 scale)	15.0 (12.0–19.0)	18.0 (15.0–20.0)	8.0 (6.0–11.0)	< 0.0001
PROMIS-CAT measures (0–100 scale)				
Pain interference	63.3 (60.3–66.9)	65.5 (62.7–67.8)	58.0 (55.8–61.5)	< 0.0001
Fatigue	63.0 (58.7–67.9)	65.7 (62.3–69.4)	56.7 (50.8–62.3)	< 0.0001
Physical function	37.8 (34.0–40.8)	35.5 (32.5–38.6)	43.2 (39.5–45.8)	< 0.0001
Sleep disturbance	59.2 (54.3–63.0)	60.8 (55.8–64.9)	55.6 (50.4–61.8)	< 0.0001

Participants with near REM: RAPID3 scores 1–3; low disease activity: RAPID3 scores 4–6; moderate disease activity: RAPID3 scores 7–12; high disease activity: RAPID3 scores 13–30. *DMARD* disease-modifying antirheumatic drug, *IQR* interquartile range, *NSAID* non-steroidal anti-inflammatory drug, *PROMIS-CAT* Patient-Reported Outcomes Measurement Information System – Computerized Adaptive Test, *RA* rheumatoid arthritis, *RAPID3* Routine Assessment of Patient Index Data 3, *REM* remission, *SD* standard deviation

^a^Statistical significance between moderate/low and high disease activity patient groups, *p* < 0.05; *t* tests were performed for continuous variables and chi square tests for categorical variables; *p* values are nominal in nature and should be interpreted in an exploratory manner

^b^Participants received prior DMARD treatment before baseline. PROMIS-CAT cut-offs for normal (score ≤ 55), low (score > 55–60), and medium (score > 60–70), and high pain interference, fatigue, and sleep disturbance (score > 70); PROMIS-CAT cut-offs for normal (score ≥ 45), low (score 40 < 45), medium (30 < 40), and high physical function (score < 30). Possible PROMIS-CAT scores ranged from 0 to 100

### Participants’ motivations and attitudes towards current RA treatment goals

**Fig. 1:**
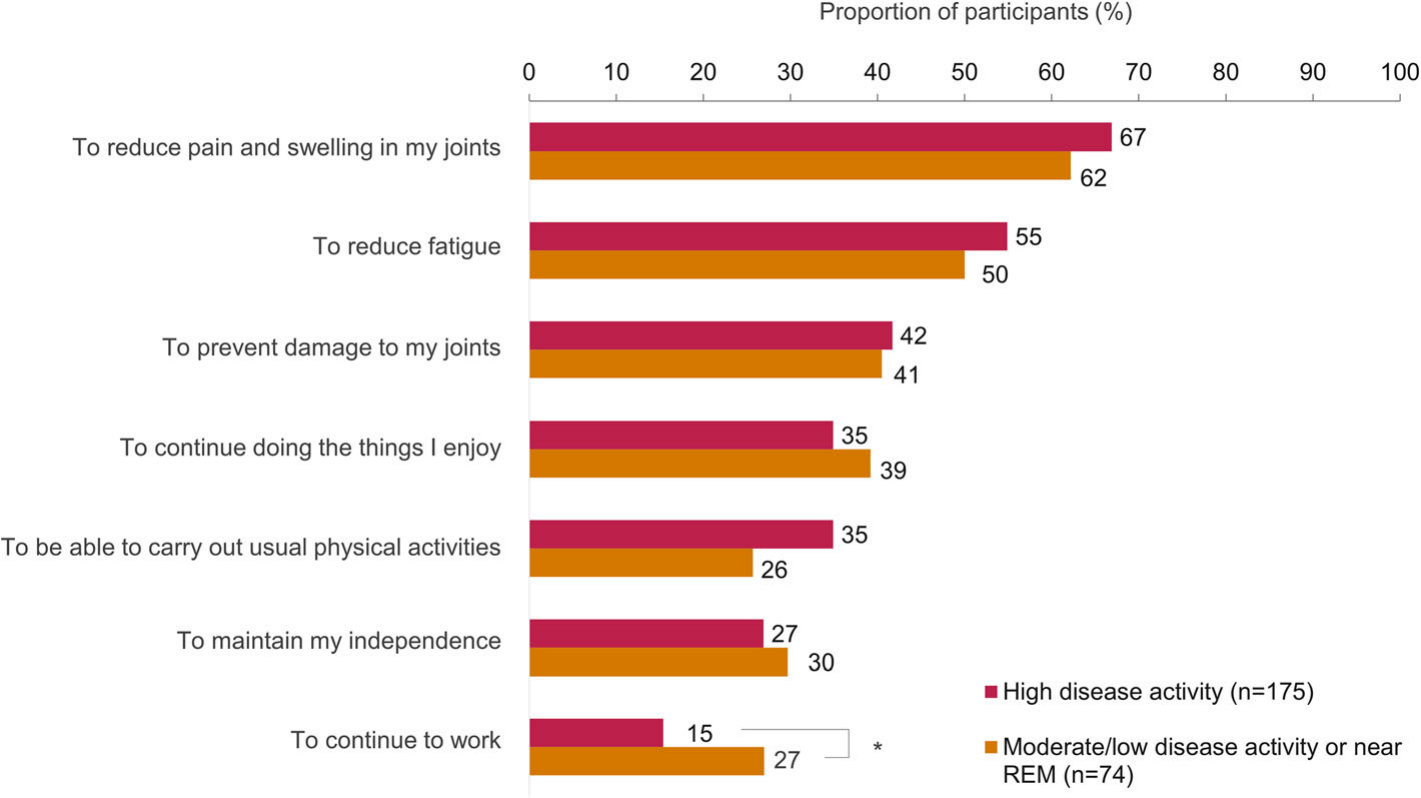
Treatment goals most important to participants in their overall management of their RA, among all surveyed participants (*N* = 249). Participants could select up to a maximum of three factors. Factors are shown if at least one group had ≥ 25% of participants who rated the factor as important. *p < 0.05; *p* values are nominal, and such be interpreted in an exploratory manner. RA rheumatoid arthritis, REM remission

**Fig. 2:**
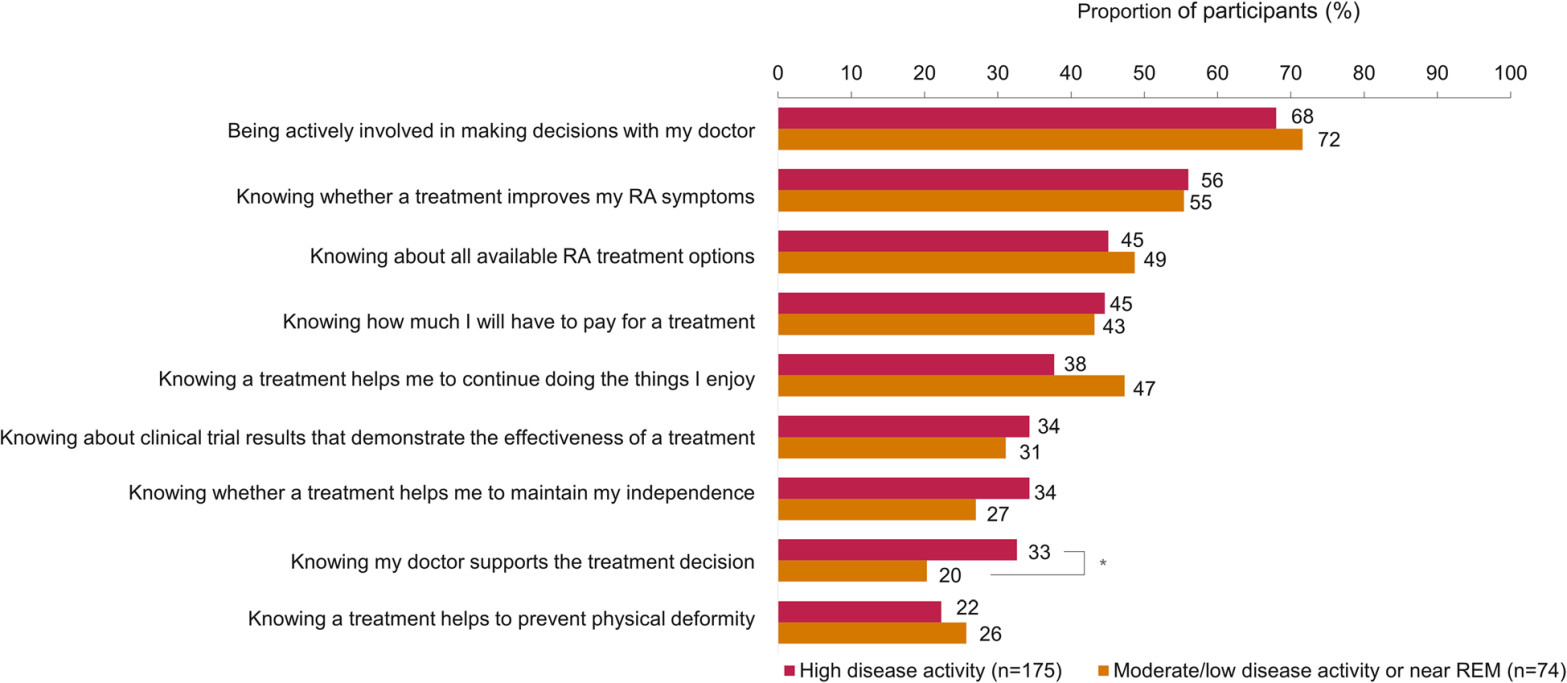
Most important factors when making treatment decisions, among all survey participants (*N* = 249). Participants could provide up to a maximum of five responses. Factors are shown if at least one group had ≥ 25% of participants who rated the factor as important. *p < 0.05; *p* values are nominal, and such be interpreted in an exploratory manner. RA rheumatoid arthritis, REM remission

Treatment goals prioritized by participants, irrespective of disease activity, were to reduce joint pain and swelling, minimize fatigue, and improve physical function (Fig. 1). Participants not in high disease activity were more likely to prioritize continued participation in work compared to participants with high disease activity (27% vs 15%, *p* = 0.03; Fig. 1). The majority of participants in high disease activity (68%) valued being actively involved in making decisions about their treatment (Fig. 2). Participants with high disease activity more frequently rated “Knowing my doctor supports the treatment decision” as important (33%), compared to participants not in high disease activity (20%) (*p* = 0.05; Fig. 2).

**Fig. 3:**
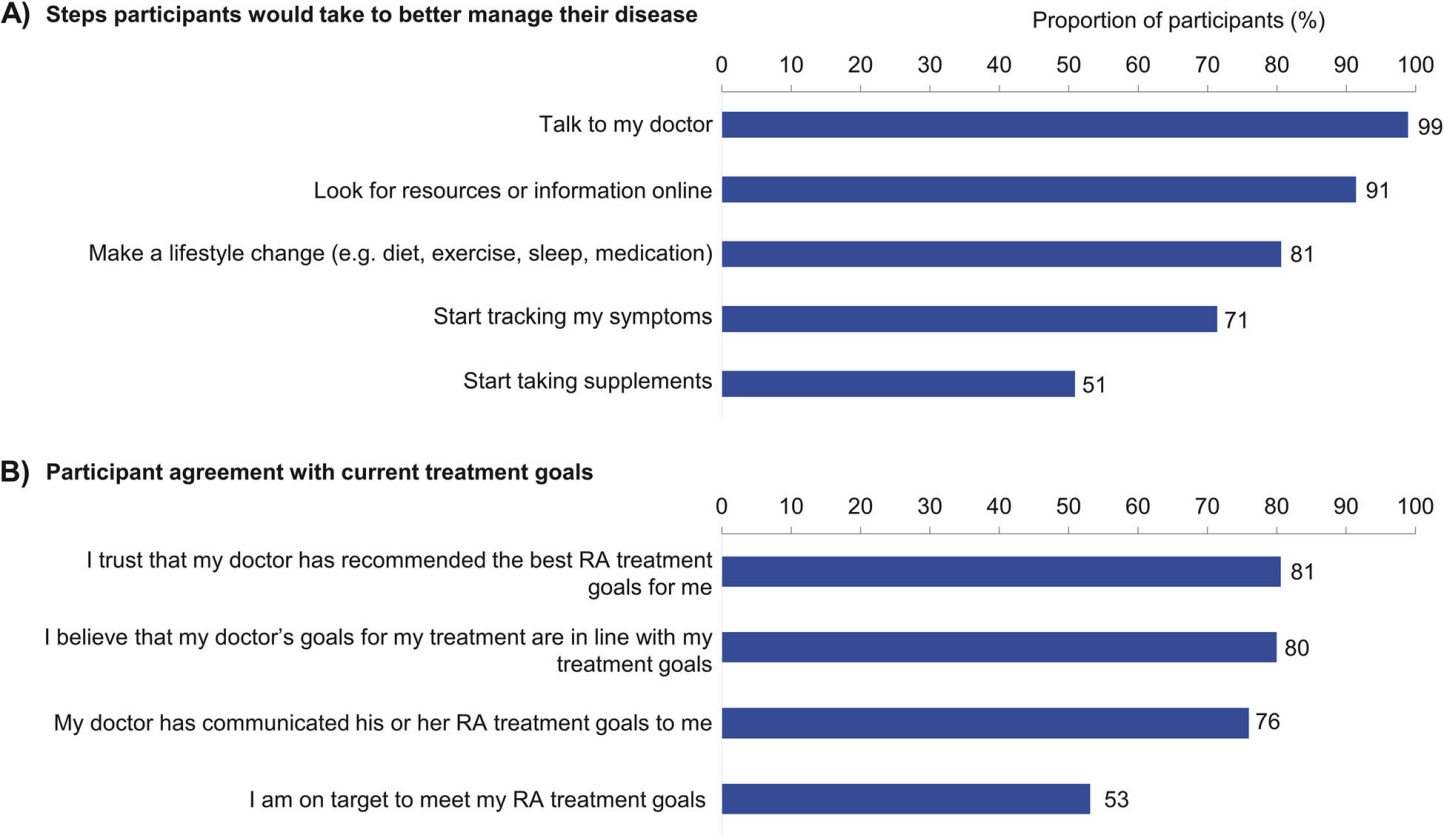
Participant motivations and attitudes towards treatment if RA symptoms were not being well managed, among participants in high disease activity (*n* = 175). **a** Participants were asked “If your RA symptoms were not being well managed, what steps would you take to better manage it?” Values reported indicate the proportion of participants who were likely or very likely to take the suggested action; top 5 actions are reported. **b** Proportion of participants who agreed or strongly agreed with the statement; all statements are shown. RA rheumatoid arthritis

Participants with high disease activity at baseline were asked to rate the likelihood that they would take specific steps if their RA symptoms were not being well-managed. The top 3 actions reported were: they would talk to their doctor (likely or very likely: 99%), look for resources or information online (likely or very likely: 91%), and make a lifestyle change, such as a change in diet, exercise, sleep, or meditation (likely or very likely: 81%) (Fig. 3a). The majority of participants with high disease activity strongly agreed or agreed that they trusted their doctor had recommended the best RA treatment goals for them (81%); however, when replying to a different statement, 20% of participants in high disease activity strongly agreed or agreed that they believed their doctor’s goals for RA treatment were not in line with their own (Fig. 3b).

### RA disease activity and treatment change

Of all surveyed participants who were offered a treatment change (39%; *n* = 98), 65 (66%) participants intensified, 15 (15%) participants scaled back, and 32 (33%) participants did not change their treatment (participants could select all responses that applied to them). Demographics, RA-related features, and current RA treatment were similar between participants who were offered a treatment change and those who were not (Additional file 1: Table S3).

**Fig. 4:**
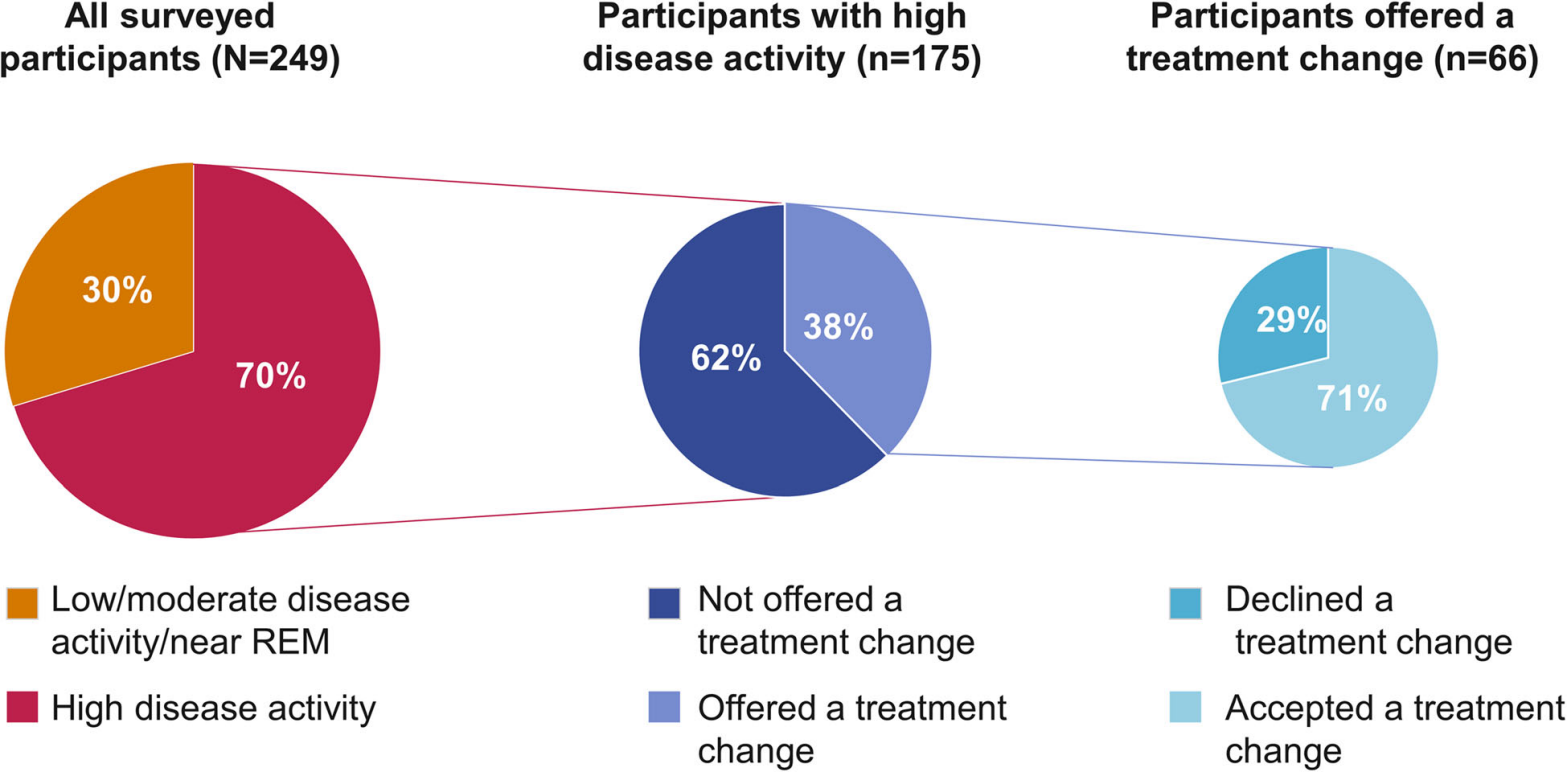
Treatment change in participants with RAPID3 high disease activity scores. Results show the proportion of participants with high disease activity, as measured by RAPID3 (> 12.0)^11^, who accepted or declined a treatment change when it was offered by their physician. RAPID3 Routine Assessment of Patient Index Data 3, REM remission

Participants were grouped according to their baseline disease activity, and among participants who had high disease activity at baseline (*n* = 175), only 66 (38%) participants were offered a treatment change at their most recent physician visit (Fig. 4). Most of these participants accepted the offered treatment change (71%). Among the subgroup of participants with persistent high disease activity (RAPID3 score > 12 at one or more additional time points in the 12 months prior to baseline; *n* = 121), only 47 (39%) were offered a treatment change at their last physician visit, and of those 35 (75%) accepted the treatment change, which is consistent with the main results. Among participants with moderate/low disease activity (*n* = 74), 32 (43%) were offered a treatment change at their most recent physician visit, 59% of whom accepted the offered treatment change.

### Factors influencing participants’ treatment decisions

**Fig. 5:**
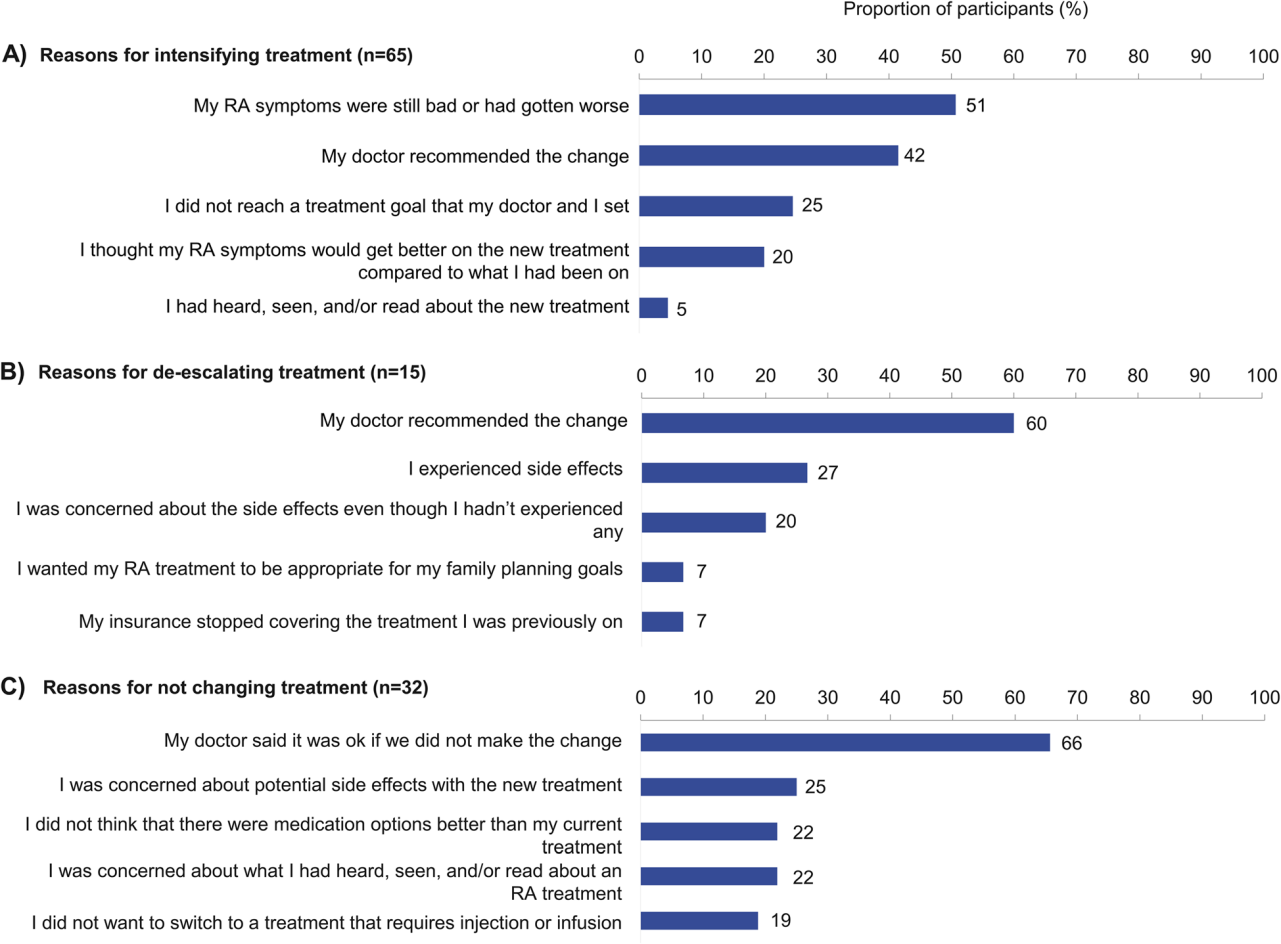
Factors influencing surveyed participants’ decisions to **a** intensify, **b** de-escalate, and **c** not change treatment, among participants who were offered a treatment change (*n* = 98). The sum of the percentages is greater than 100% as participants could select more than one response, up to a maximum of three factors. Participants were asked “When thinking about your last treatment change, which of the factors below had the strongest influence on your decision to change?” in reference to **a** more intensive treatment, **b** de-escalated treatment, or **c** no change to treatment. The top 5 factors are shown. RA rheumatoid arthritis

Over half of all surveyed participants who intensified their treatment (33/65; 51%) did so because their symptoms remained bad or worsened, whereas only 25% (16/65) changed because they did not reach pre-defined treatment goals (Fig. 5a). Physician recommendation was a major reason given by participants for intensifying (42%; Fig. 5a) or scaling back treatment (60%; Fig. 5b). The most common reason (66%) for deciding not to change treatment was the rheumatologist’s satisfaction with the current therapy; participant concern related to potential side effects of the new therapy was much less common (25%; Fig. 5c).

### Participants’ perception of disease activity in relation to RAPID3 score and PROMIS measurements

**Fig. 6:**
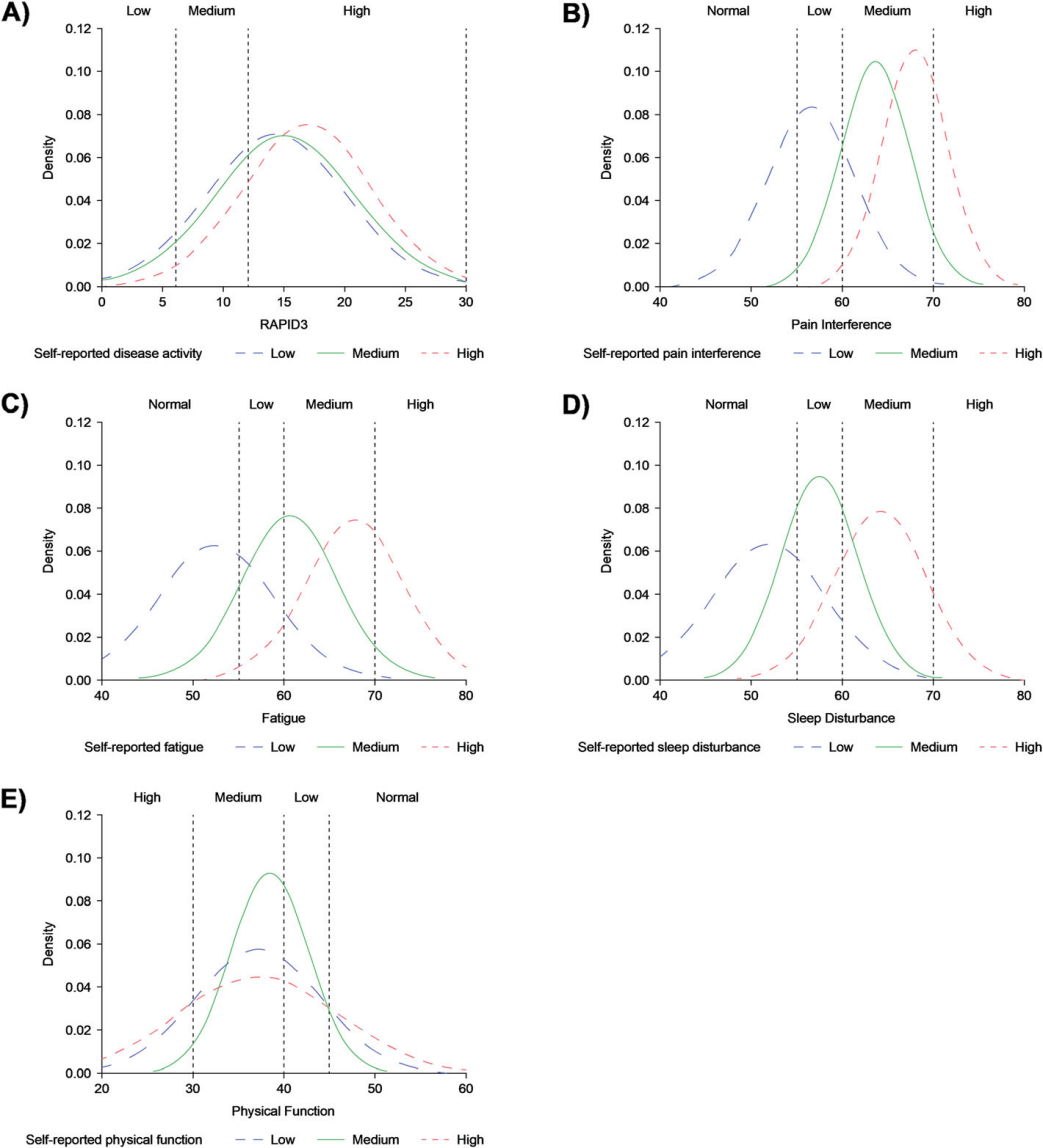
Comparison of participants’ self-perception of **a** disease activity, **b** pain interference, **c** fatigue, **d** sleep disturbance, and **e** physical function compared to RAPID3 (**a**) or PROMIS-CAT scores (**b**–**e**). **a** Participants were asked “How would you describe overall RA disease activity, on average, over the past 7 days?” Responses were compared with participants’ RAPID3 scores. **b** Participants were asked “How would you describe your pain, on average, over the past 7 days?” Responses were compared with participants’ PROMIS-CAT scores for pain interference. **c** Participants were asked “How would you describe your fatigue on average, over the past 7 days?” Responses were compared with participants’ PROMIS-CAT scores for fatigue. **d** Participants were asked “How would you describe your difficulty sleeping on average, over the past 7 days?” Responses were compared with participants’ PROMIS-CAT scores for sleep disturbance. **e** Participants were asked “How would you describe your ability to carry out physical activity on average, over the past 7 days?” Responses were compared with participants’ PROMIS-CAT scores for physical function. Vertical dashed lines indicate RAPID3 cut-offs for low (score > 3 ≤ 6), medium (score > 6 ≤ 12), and high disease activity (score > 12 ≤ 30); PROMIS-CAT cut-offs for normal (score ≤ 55), low (score > 55–60), medium (score > 60–70), and high pain interference, fatigue, and sleep disturbance (score > 70); PROMIS-CAT cut-offs for normal (score ≥ 45), low (score 40 < 45), medium (30 < 40), and high physical function (score < 30). Possible PROMIS-CAT scores ranged from 0 to 100. PROMIS-CAT Patient-Reported Outcomes Measurement Information System – Computerized Adaptive Tests; RAPID3 Routine Assessment of Patient Index Data 3

Patients’ treatment decisions may also be influenced by their perception of their disease activity. When participants were asked to self-rate their disease activity, most of those who rated their disease activity as low or moderate had high RAPID3 disease activity (weighted kappa [95% CI]: 0.05 [− 0.01, 0.11]; Fig. 6a). By contrast, there was a moderate agreement between participants’ self-rated levels of pain interference (weighted kappa [95% CI]: 0.44; [0.35, 0.52]), fatigue (weighted kappa [95% CI]: 0.36; [0.28, 0.44]), and sleep disturbance (weighted kappa [95% CI]: 0.25; [0.19, 0.31]) and the corresponding PROMIS measurement (Fig. 6b–d). There was low agreement between participants’ self-rated ability to carry out physical activities and the corresponding PROMIS measurement (weighted kappa [95% CI]: 0.02; [− 0.08, 0.13]; Fig. 6e).

## Discussion

This study showed that despite contemporary T2T recommendations in RA, only 38% of participants with high disease activity, as measured by RAPID3, were offered a treatment change at their most recent visit with their rheumatologist. These observations were consistent for participants who had persistent high disease activity, which confirmed that failure of physicians to offer a treatment change was not simply an artifact of short-term worsening (flare) of RA. The majority of participants cited their doctor’s opinion (rather than their own) as the key influential factor in the treatment decision, whether it was for intensifying treatment (42%), scaling back treatment (60%), or not making a treatment change (66%). A key part of the T2T approach involves the rheumatologist and patient setting treatment targets together as a shared decision, which is not apparent from these data. We also found that agreement between RAPID3 categories and participants’ own assessment of their disease activity was only modest, suggesting that the RAPID3 may not be very informative when setting treatment goals from a patient’s perspective. A higher level of agreement was observed between participant’s self-rated level of pain, fatigue, and sleep disturbance and the corresponding PROMIS measurement; however, a lower level of agreement was observed for physical function. The results of our study illustrate the importance of considering patient perspectives in T2T recommendations and the need to tailor interventions with a patient-centric focus, rather than solely a physician focus.

Findings from this study also show that participants adopted a passive approach when considering intensification of their treatment and reflect previous observations that patients commonly looked to their doctor to initiate a treatment change or self-manage their symptoms until they can no longer cope, despite experiencing high disease activity [13, 14]. Similarly, over half of participants who responded to a previous survey (*n* = 6135) expressed satisfaction with their disease control such that they did not need new medications; however, many of these patients had moderate or high disease activity [15]. Patients in general would benefit from education on T2T guidelines, the importance of their involvement in setting treatment targets and the importance of achieving low disease activity or remission on their overall, long-term health. This may increase patient involvement in decision-making and setting treatment goals, which may improve their short- and long-term outcomes. While we show that most participants would speak to their rheumatologist if their symptoms were not well controlled (99%), online resources were also judged to be very important by most participants (91%). Current online resources providing information about RA to patients may not be fit for purpose as they are often outdated and do not cover all of patients’ educational requirements [16]. Patients who considered their illness as episodic or progressively deteriorating reported needing more support with self-management of their condition than patients who perceived their disease as stable [17]. Additionally, the readability of online resources designed for patients with RA can vary widely and most use language that is more complex than the standard 6^th^ grade reading level recommended for patient materials [18].

We found that participants seldom changed RA therapies because they failed to reach treatment goals; instead, treatment was more commonly changed due to continued severe, or even worsening, RA symptoms. This concept of loss aversion or avoidance is expected, since worsening (or fear of worsening) health status is a greater motivator to change than optimism that a new therapy might result in low disease activity or remission, and feeling better as a result [19]. A patient-centric T2T intervention needs to consider this important motivation in human decision-making. Indeed, setting patients’ expectations to strive for disease remission at the time of RA diagnosis may help to facilitate subsequent treatment changes. Knowing that their doctor supported their treatment decision was considered important by fewer participants (29%) compared to those who placed importance on being actively involved in making treatment decisions (69%). This suggests that participants are more concerned about making treatment decisions collaboratively with their doctor, including learning about treatment options, costs, and potential impact on their day-to-day life, than whether their doctor approves of their final decision.

In the present study, the established RAPID3 categories of disease activity and the PROMIS physical function component did not agree well with the corresponding participants’ classification of their present health state. This misalignment may be expected as self-reported disease activity is highly subjective and may be a difficult concept for patients to describe. Patients may perceive high disease activity as the worst disease status that they have ever experienced, which may lead them to underreport the severity of their disease. Furthermore, physical function may be difficult for people with RA to assess, particularly as their expectations and perspective of “normal function” may change dramatically as their disease progresses. By contrast, the PROMIS measurements of pain interference, fatigue, and sleep disturbance were much better aligned with participants’ perception of these outcomes. These findings suggest that patients’ illness perceptions may not be aligned with some of the PRO metrics that rheumatologists most commonly perceive as being patient-focused (e.g., RAPID3). These results also indicate that there is a need to re-evaluate how the results of the RAPID3, used as a measure of disease activity, are communicated to patients, along with the T2T recommended composite measurements of disease activity. This is particularly relevant as patient representation among the experts involved in the 2009 and 2014 International Task Force discussions was low [2, 20]. Setting patients’ expectations regarding RA disease targets, and what it means to be doing “well,” needs further study. A more personalized approach may be necessary rather than attaining low disease activity or remission by traditional composite RA metrics like the RAPID3, CDAI, or DAS28 [11, 21, 22]. The current T2T guidance uses an algorithmic approach for making treatment decisions, so it may be appropriate for future approaches to require more input from both patients and rheumatologists.

Among the limitations of this study was that this cross-sectional survey required participants to recall whether they were offered a recent treatment change, which could be subject to misclassification; to minimize this bias, participants were only asked to recall their most recent physician visit. Participants’ RA diagnosis was self-reported, but the fact that participants had to be current or past biologic or DMARD users with an RA-specific treatment and to report on current care by a U.S. rheumatologist, contributed to increased specificity [23]. Disease activity was measured only by RAPID3 and not by rheumatologist-derived measures such as CDAI or DAS28. Thus, failure of the rheumatologist to offer a treatment change may have been appropriate and in line with current T2T guidelines. These observations highlight the need to tailor measurements collected by rheumatologists to ensure that patient’s goals and expectations are considered when assessing the need for treatment changes. Furthermore, patients with long-standing RA can have complex comorbidities, requiring the prescription of concomitant medications, which may have influenced physician’s treatment decisions as well. RAPID3 is one of the instruments permitted for the measurement of RA disease activity in the T2T guidelines, and indeed rheumatologists commonly only use the RAPID3 in their clinical practice [24]. Since various quality of care metrics in RA do not require physician input, these results reflect the realities of current RA clinical care. An additional limitation is that patients who elect to join an online research registry, and those who responded to this survey, may not be representative of all patients with RA, and thus, these data may not be generalizable to RA patients engaged in other settings.

## Conclusions

A large proportion of the surveyed participants had high disease activity, as measured by RAPID3, but only about one third of these participants were offered a treatment change. Participants commonly accepted their rheumatologist’s opinion that a treatment change was not required, suggesting that achieving their doctor’s treatment goals was considered more important than their own personal targets. Patients should be educated on the short- and long-term benefits of achieving disease control and encouraged to utilize treatment change as the means to achieving this goal. Traditional RA disease activity measures (e.g., RAPID3) may not be suitable to assess achievement of patients’ own treatment goals. Setting patient-centric goals, using metrics which are relevant to patients, are just as important to personalizing RA care and improving outcomes as reaching treatment targets of remission or LDA.

## Supplementary information

**Additional file 1: Table S1.** All questions within the ArthritisPower custom, online survey**. Table S2.** Baseline participant demographics and disease characteristics for all ArthritisPower RA participants compared to the surveyed participants in this study. **Table S3.** Baseline participant demographics and disease characteristics for participants offered/not offered a treatment change (*N* = 249)

## Data Availability

The data that support the findings of this study are available from the corresponding author, KG, upon request.

## Abbreviations

ANOVA: Analysis of variance
CDAI: Clinical Disease Activity Index
DMARD: Disease-modifying antirheumatic drug
DAS: Disease activity score
DAS28: 28-Joint Disease Activity Score
NSAID: Non-steroidal anti-inflammatory drug
LDA: Low disease activity
PRO: Patient-reported outcome
PROMIS-CAT: Patient-Reported Outcomes Measurement Information System – Computerized Adaptive Tests
RA: Rheumatoid arthritis
RAPID3: Routine Assessment of Patient Index Data 3
REM: Remission
SD: Standard deviation
SDAI: Simplified disease activity index
T2T: Treat-to-target
