# Supplementary material for: Barriers to treatment optimization and achievement of patients’ goals: perspectives from people living with rheumatoid arthritis enrolled in the ArthritisPower registry
Source: Arthritis Res Ther. 2020 Jan 7;22:4. doi: 10.1186/s13075-019-2076-7 (PMC6947932; doi:10.1186/s13075-019-2076-7)
Supplement: Supplementary file 1 — Additional file 1: Table S1. All questions within the ArthritisPower custom, online survey. Table S2. Baseline participant demographics and disease characteristics for all ArthritisPower RA participants compared to the surveyed participants in this study. Table S3. Baseline participant demographics and disease characteristics for participants offered/not offered a treatment change (N = 249) [file 13075_2019_2076_MOESM1_ESM.docx]

**Barriers to Treatment Optimization and Achievement of Patients’ Goals: Perspectives from People Living with Rheumatoid Arthritis Enrolled in the ArthritisPower Registry**

Kelly Gavigan,^1^ W. Benjamin Nowell,^1^ Mylene S. Serna,^2^ Jeffrey L. Stark,^2^ Mohamed Yassine,^2^ Jeffrey R. Curtis^3^

^1^Global Healthy Living Foundation, Upper Nyack, New York, USA; ^2^UCB Pharma, Smyrna, Georgia, USA; ^3^University of Alabama at Birmingham, Birmingham, Alabama, USA

SUPPLEMENTARY DATA

**Table S1.** All questions within the ArthritisPower custom, online survey

| **Survey questions and potential responses** | |
| --- | --- |
| ***Which goals are the most important to you in the overall management of your RA?***  ***Please select up to three factors:*** | |
| To reduce pain and swelling in my joints | |
| To reduce fatigue | |
| To prevent damage to my joints | |
| To continue to do the things I enjoy (e.g. hobbies, gardening, sports, playing an instrument, playing with kids/grandkids) | |
| To be able to carry out usual physical activities (e.g. cooking, cleaning, bathing, dressing) | |
| To maintain my independence | |
| To continue to work | |
| To participate in social activities with friends and family | |
| To improve emotional wellbeing | |
| ***Sources of information about RA:*** | |
| My rheumatologist | |
| Online educational websites | |
| Online support groups or blogs | |
| Print materials like pamphlets and books | |
| Other people I know diagnosed with RA | |
| My primary care physician | |
| Pharmacist | |
| Advertising from pharmaceutical companies | |
| Other healthcare providers | |
| Family or friends | |
| Other | |
| I have not received information about RA [exclusive choice] | |
| ***Aspects of RA treatment or care for which more information is needed:*** | |
| Long-terms effects of treatment | |
| Foods/diet that improve symptoms | |
| Ways to improve my ability to do usual physical activities | |
| Behaviours or activities that improve symptoms (e.g. meditation, exercise) | |
| Treatment options available to me | |
| How to access online or in-person support groups | |
| I don’t want/need additional information about my treatment or care | |
| ***Most important factors for RA treatment decision making:*** | |
| Being actively involved in making decisions with my doctor about a treatment | |
| Knowing whether a treatment improves my RA symptoms | |
| Knowing about all available RA treatment options | |
| Knowing how much I will have to pay for a treatment | |
| Knowing a treatment helps me to continue doing things I enjoy (e.g. hobbies, gardening, sports, playing an instrument, playing with kids/grandkids) | |
| Knowing about clinical trial results that demonstrate the effectiveness of a treatment on RA symptoms and disease progression | |
| Knowing whether a treatment helps me to maintain my independence and not rely on other too much | |
| Knowing my doctor supports the treatment decision | |
| Knowing a treatment helps to prevent physical deformity | |
| Knowing whether a treatment helps improve my usual physical activities | |
| Knowing how a treatment might help me reach my treatment goals | |
| Knowing what happens if my RA is not adequately treated | |
| Knowing whether a treatment helps me continue working | |
| Knowing the impact that a treatment might have on my family life | |
| Hearing from another patient like me who took the same treatment | |
| Knowing the impact that a treatment might have on my family planning goals (i.e., conceiving a child, pregnancy or breastfeeding) | |
| Getting the advice of a friend and/or loved one about a treatment | |
| ***When thinking about your last treatment change, which of the factors below had the strongest influence on your decision to change?***  ***Please select up to three factors:*** | |
| My doctor recommended the change | |
| My RA symptoms were still bad or had gotten worse on the treatment I was previously on | |
| I did not reach a treatment goal (i.e. lower disease activity score) that my doctor and I had set | |
| I thought my RA symptoms would get better on the new treatment compared to what I had been on | |
| I had heard, seen and/or read about the new treatment | |
| Someone I know is/was on the new treatment and had a good experience | |
| I did not think I could refuse to make a treatment change suggested by my doctor | |
| My insurance plan offered lower costs to me for the treatment that I changed to | |
| I experienced side effects from the treatment I was previously on | |
| I was concerned about the side effects of the treatment even though I hadn’t experienced any | |
| I wanted my RA treatment to be appropriate for my family planning goals (i.e., conceiving a child, pregnancy or breastfeeding) | |
| My insurance stopped covering the treatment I was previously on | |
| I wanted to change the way my RA treatment is given (e.g., tablets, injection, or infusion) | |
| My insurance plan covers the treatment that I changed to | |
| ***When thinking about your most recent decision to NOT change treatments, which factors had the strongest influence on your decision? Please select up to three factors:*** | |
| My doctor said it was ok if we did not make the change | |
| I was concerned about potential side effects with the new treatment | |
| I did not think there were medication options better than my current treatment | |
| I was concerned about what I had heard, seen and/or read about an RA treatment | |
| I did not want to switch to a treatment that requires injection or infusion | |
| I could not afford the cost of a different treatment | |
| I felt satisfied with the current state of my RA symptoms | |
| I did not want to deal with getting approval from my insurance company for a different treatment | |
| I was concerned about an increase in RA symptoms with the new treatment | |
| I did not want to increase the number of medications I am taking | |
| I was concerned about drug interactions with the new treatment | |
| I did not want to fill a new prescription so soon after refilling the prescription for my current treatment | |
| I was not given enough time to make a decision about changing my RA treatment | |
| I wanted my RA treatment to be appropriate for my family planning goals (i.e., conceiving a child, pregnancy or breastfeeding) | |
| I did not want to decrease the number of medications I am taking | |
| I did not want to make a lifestyle change (e.g., no longer drinking alcohol, going in for regular infusions) | |
| ***Which healthcare provider do you see most often for the treatment and care of your RA?*** | |
| Rheumatologist | |
| General practitioner/family doctor | |
| Nurse practitioner | |
| Physician’s assistant | |
| ***How often do you visit your [healthcare provider indicated above]?*** | |
| Every 1–3 months | |
| Every 4–6 months | |
| Yearly | |
| I don’t see my doctor regularly | |
| ***Which other healthcare providers have you seen for the treatment and care of your RA?*** | |
| General practitioner/family doctor | |
| Rheumatologist | |
| Pharmacist | |
| Nurse practitioner | |
| Allied health professionals (i.e. dietitian/nutritionist, physical therapist, clinical psychologist, medical assistant, occupational therapist, social worker | |
| Physician’s assistant | |
| Nurse/registered nurse | |
| ***How would you describe your overall RA disease activity over the past 7 days?*** | |
| None/low | |
| Medium | |
| High | |
| ***How would you describe your pain over the past 7 days?*** | |
| None/low | |
| Medium | |
| High | |
| ***How would you describe your ability to carry out your usual physical activity over the past 7 days?*** | |
| None/low | |
| Medium | |
| High | |
| ***How would you describe your fatigue over the past 7 days?*** | |
| None/low | |
| Medium | |
| High | |
| ***How would you describe your difficulty sleeping over the past 7 days?*** | |
| None/low | |
| Medium | |
| High | |
| ***What was the effect of your treatment change on your RA symptoms? [Participants who had changed treatment]*** | |
| I did not notice a change in RA symptoms | |
| My RA symptoms got better | |
| My RA symptoms got worse | |
| ***Reasons why a treatment change was not discussed at your last visit? [Participants who were not offered a treatment change]*** | |
| My provider is satisfied with my current treatment | |
| I am satisfied with my current treatment and I have told my provider this | |
| Other | |
| I do not discuss treatment options with my provider | |
| ***What concerns do you have about your ability to see your treating doctor?*** | |
| I don’t have concerns about my ability to see a doctor to treat my RA [exclusive choice] | |
| It takes me too long to get an appointment with my doctor | |
| I don’t have enough doctors to choose from for my care | |
| I don’t have enough time with my doctor during a visit | |
| It costs me too much to see a doctor | |
| It takes me too long to travel to my doctor | |
| I have to wait too long in the waiting room | |
| I don’t have transportation to visit my doctor | |
| ***Participant motivations to change treatment among participants with high disease activity:*** | |
| I trust that my doctor has recommended the best RA treatment goals for me | Strongly agree/ Agree/ Neither agree nor disagree/ Disagree/ Strongly disagree |
| I believe that my doctor’s goals for my RA treatment are in line with my treatment goals | Strongly agree/ Agree/ Neither agree nor disagree/ Disagree/ Strongly disagree |
| My doctor has communicated his or her RA treatment goals to me | Strongly agree/ Agree/ Neither agree nor disagree/ Disagree/ Strongly disagree |
| I am on target to meet my RA treatment goals | Strongly agree / Agree/ Neither agree nor disagree/ Disagree/ Strongly disagree |
| ***If your RA symptoms were not being well managed, what steps would you take to better manage it? Please indicate the likelihood that you would complete the following actions:*** | |
| Talk to my doctor | Very likely to do/ Likely to do/ Neither likely nor unlikely to do/ Unlikely to do/ Very unlikely to do |
| Look for resources or information online | Very likely to do/ Likely to do/ Neither likely nor unlikely to do/ Unlikely to do/ Very unlikely to do |
| Make a lifestyle change (e.g. diet, exercise, sleep, meditation) | Very likely to do/ Likely to do/ Neither likely nor unlikely to do/ Unlikely to do/ Very unlikely to do |
| Start tracking my symptoms | Very likely to do/ Likely to do/ Neither likely nor unlikely to do/ Unlikely to do/ Very unlikely to do |
| Start taking supplements | Very likely to do/ Likely to do/ Neither likely nor unlikely to do/ Unlikely to do/ Very unlikely to do |
| Talk to other patients | Very likely to do/ Likely to do/ Neither likely nor unlikely to do/ Unlikely to do/ Very unlikely to do |
| Change doctors | Very likely to do/ Likely to do/ Neither likely nor unlikely to do/ Unlikely to do/ Very unlikely to do |
| Talk to my pharmacist | Very likely to do/ Likely to do/ Neither likely nor unlikely to do/ Unlikely to do/ Very unlikely to do |
| ***Please indicate how easy or hard it is for you to access the following:*** | |
| Scheduling an appointment with a/my rheumatologist | Very easy/ Easy/ Neither easy nor hard/ Hard/ Very hard |
| Getting my RA prescription(s) filled on time | Very easy/ Easy/ Neither easy nor hard/ Hard/ Very hard |
| Getting a referral to a specialist who is not my regular prescribing physician | Very easy/ Easy/ Neither easy nor hard/ Hard/ Very hard |
| Getting my RA treatment approved by my insurance company | Very easy/ Easy/ Neither easy nor hard/ Hard/ Very hard |
| Scheduling an appointment to have my treatment administered if I take an infusion or injection administered by a healthcare provider | Very easy/ Easy/ Neither easy nor hard/ Hard/ Very hard |
| Having a choice in rheumatologists in my area | Very easy/ Easy/ Neither easy nor hard/ Hard/ Very hard |
| Being able to afford the cost of my RA treatment | Very easy/ Easy/ Neither easy nor hard/ Hard/ Very hard |
| ***Comparison of attitudes among participants with high disease activity*** | |
| I know how my RA treatment is supposed to be taken | Strongly agree/Agree/ Neither agree nor disagree/ Disagree/ Strongly disagree |
| I know what to do if I miss a dose of my RA treatment (e.g., whether to make up a dose later) | Strongly agree/Agree/ Neither agree nor disagree/ Disagree/ Strongly disagree |
| I know what the possible side effects of my RA treatment are | Strongly agree/Agree/ Neither agree nor disagree/ Disagree/ Strongly disagree |
| Tracking my RA helps me know when my symptoms have changed | Strongly agree/Agree/ Neither agree nor disagree/ Disagree/ Strongly disagree |
| Tracking my RA helps me see whether a treatment or lifestyle change has had an  impact on my symptoms | Strongly agree/Agree/ Neither agree nor disagree/ Disagree/ Strongly disagree |
| I understand the risks of under-treating my RA | Strongly agree/Agree/ Neither agree nor disagree/ Disagree/ Strongly disagree |
| Tracking my RA helps improve my communication with my doctor | Strongly agree/Agree/ Neither agree nor disagree/ Disagree/ Strongly disagree |
| It upsets me that the RA medication(s) I have been prescribed can cause troublesome side effects (e.g., headache, upset stomach) | Strongly agree/Agree/ Neither agree nor disagree/ Disagree/ Strongly disagree |
| I know how my RA treatment works in my body to treat RA | Strongly agree/Agree/ Neither agree nor disagree/ Disagree/ Strongly disagree |
| Tracking my RA helps me know when I should change my RA treatment | Strongly agree/Agree/ Neither agree nor disagree/ Disagree/ Strongly disagree |
| It upsets me that the RA medication(s) I have been prescribed have a very rare chance of causing a serious infection or permanent condition | Strongly agree/Agree/ Neither agree nor disagree/ Disagree/ Strongly disagree |
| I am worried that the RA treatment I have been prescribed will hurt my health | Strongly agree/Agree/ Neither agree nor disagree/ Disagree/ Strongly disagree |
| It frustrates me to think that I will have to take RA medication(s) for the rest of my life | Strongly agree/Agree/ Neither agree nor disagree/ Disagree/ Strongly disagree |
| It is easy for me to stay informed about RA treatment options | Strongly agree/Agree/ Neither agree nor disagree/ Disagree/ Strongly disagree |
| Tracking my RA helps me know when I should see my doctor | Strongly agree/Agree/ Neither agree nor disagree/ Disagree/ Strongly disagree |
| Tracking my RA helps others (e.g., family and friends) understand my condition | Strongly agree/Agree/ Neither agree nor disagree/ Disagree/ Strongly disagree |
| It upsets me that the RA medication(s) I have been prescribed can affect the way  I look | Strongly agree/Agree/ Neither agree nor disagree/ Disagree/ Strongly disagree |
| I get frustrated taking my RA medication(s) because I have to plan my life around it | Strongly agree/Agree/ Neither agree nor disagree/ Disagree/ Strongly disagree |
| Tracking my RA distracts me from doing other things I want to do | Strongly agree/Agree/ Neither agree nor disagree/ Disagree/ Strongly disagree |
| I don’t like taking my RA medication(s) because it reminds me that I have RA | Strongly agree/Agree/ Neither agree nor disagree/ Disagree/ Strongly disagree |
| As long as I am feeling healthy, missing my RA treatment from time to time is OK | Strongly agree/Agree/ Neither agree nor disagree/ Disagree/ Strongly disagree |

Table S2. Baseline participant demographics and disease characteristics for all ArthritisPower RA participants compared to the surveyed participants in this study

|  | ArthritisPower RA participants^a^ (N=5,541) | All survey  participants  (N=249) |
| --- | --- | --- |
| Age, years | 50.9 (10.9) | 51.7 (11.0) |
| Females, n (%) | 5136 (92.7) | 229 (92.0) |
| Ethnicity, white, n (%) | 4909 (88.6) | 225 (90.4) |
| Time since diagnosis, years (SD)^b^ | - | 11.0 (9.5) |
| Some college education or above, n (%)^b^ | - | 215 (86.4) |
| Full-time employment, n (%) | 1579 (28.5) | 83 (33.3) |
| Private insurance, n (%) | 2216 (40.0) | 154 (61.9) |
| Current RA Therapy, n (%)^b^ | | |
| Non-biologic DMARDs only | - | 72 (28.9) |
| Biologic DMARDs | - | 150 (60.2) |
| Steroid/NSAID/other/no treatment^c^ | - | 27 (10.8) |
| Patient reported outcomes, median (IQR) | | |
| RAPID3 (0–30 scale) | 16.0 (12.0-20.0) | 15.0 (12.0–19.0) |
| PROMIS-CAT measures (0–100 scale) | | |
| Pain interference | 64.7 (61.5-68.4) | 63.3 (60.3–66.9) |
| Fatigue | 64.8 (60.5-71.2) | 63.0 (58.7–67.9) |
| Physical function | 36.6 (31.5-40.2) | 37.8 (34.0–40.8) |
| Sleep disturbance | 59.9 (54.3-65.3) | 59.2 (54.3–63.0) |

^a^Data as of December 15, 2017. ^b^Data collected for study participants only. ^c^Participants received prior DMARD treatment before baseline. PROMIS-CAT cut-offs for normal (score ≤55), low (score >55–60), and medium (score >60–70), and high pain interference, fatigue, and sleep disturbance (score >70); PROMIS-CAT cut-offs for normal (score ≥45), low (score 40 <45), medium (30 <40), and high physical function (score <30). Possible PROMIS-CAT scores ranged from 0–100. DMARD: disease modifying anti-rheumatic drug; IQR: interquartile range; NSAID: non-steroidal anti-inflammatory drug; PROMIS-CAT: Patient-Reported Outcomes Measurement Information System – Computerized Adaptive Test; RA: rheumatoid arthritis; RAPID3: Routine Assessment of Patient Index Data 3; SD: standard deviation.

Table S3. Baseline participant demographics and disease characteristics for participants offered/not offered a treatment change (N=249)

| Mean (SD) unless otherwise specified | Survey participants (N=249) | Offered a treatment change (n=98) | Not offered a treatment change (n=151) | p value |
| --- | --- | --- | --- | --- |
| Age, years | 51.7 (11.0) | 50.8 (11.5) | 52.3 (10.6) | 0.27 |
| Females, n (%) | 229 (92.0) | 89 (90.8) | 140 (92.7) | 0.59 |
| Ethnicity, white, n (%) | 225 (90.4) | 89 (90.8) | 136 (90.1) | 0.84 |
| Time since diagnosis, years | 11.0 (9.5) | 10.2 (8.3) | 11.6 (10.2) | 0.27 |
| Some college education or above, n (%) | 215 (86.4) | 84 (85.7) | 131 (86.8) | 0.82 |
| Full-time employment, n (%) | 83 (33.3) | 28 (28.6) | 55 (36.4) | 0.20 |
| Private insurance, n (%) | 154 (61.9) | 58 (59.2) | 96 (63.6) | 0.49 |
| Current RA therapy, n (%) | | | | |
| Non-biologic DMARDs only | 72 (28.9) | 31 (31.6) | 41 (27.2) | 0.45 |
| Biologic DMARDs | 150 (60.2) | 57 (58.2) | 93 (61.6) | 0.59 |
| Steroid/NSAID/other/no treatment^a^ | 27 (10.8) | 10 (10.2) | 17 (11.3) | 0.79 |
| Patient-reported outcomes, median (IQR) | | | | |
| RAPID3 (0–30 scale) | 15.0 (12.0–19.0) | 15.0  (12.0–19.0) | 15.0  (12.0–19.0) | 0.72 |
| PROMIS-CAT measures (0–100 scale) | | | | |
| Pain interference | 63.3  (60.3–66.9) | 63.8  (61.4–66.9) | 62.9  (59.1–66.9) | 0.25 |
| Fatigue | 63.0  (58.7–67.9) | 63.7  (58.7–68.8) | 62.6  (57.5–67.9) | 0.27 |
| Physical function | 37.8  (34.0–40.8) | 37.1  (33.3–41.3) | 38.2  (34.6–40.6) | 0.17 |
| Sleep disturbance | 59.2  (54.3–63.0) | 60.6  (54.3–63.0) | 58.9  (54.3–63.0) | 0.30 |

^a^Participants had received prior DMARD treatment. DMARD: disease modifying anti-rheumatic drug; IQR: interquartile range; NSAID: non-steroidal anti-inflammatory drug; PROMIS-CAT: patient-reported outcomes measurement information system – computerized adaptive test; RA: rheumatoid arthritis; RAPID3: routine assessment of patient index data 3; REM: remission; SD: standard deviation.
